# Differences in hypertension between blacks and whites: an overview

**Published:** 2007

**Authors:** Jane Lindhorst, Nicole Alexander, Juliet Blignaut, Brian Rayner

**Affiliations:** Division of Hypertension, Department of Medicine, Groote Schuur Hospital and University of Cape Town; Division of Hypertension, Department of Medicine, Groote Schuur Hospital and University of Cape Town; Division of Hypertension, Department of Medicine, Groote Schuur Hospital and University of Cape Town

## Abstract

Hypertension is more prevalent and severe in urban black populations compared to whites, and is associated with a greater degree of target-organ damage for any given blood pressure level. In general, compared to whites, blacks respond well to diuretics and calcium channel blockers and less well to β-blockers and ACE inhibitors. The exact mechanisms that contribute to differences in blood pressure between blacks and whites are still not fully understood, given the multi-factorial aetiology of essential hypertension. Various lines of evidence suggest black patients are more salt sensitive than whites, which is due to a tendency to retain sodium in the kidney. Inherent differences in ionic transport mechanisms, the renal epithelial sodium channel, the renin-angiotensin-aldosterone system and vasoactive substances may be a partial explanation, but analysis is compounded by disparate socio-economic conditions between blacks and whites. At present, there is no complete explanation for these differences and further research is required.

## Summary

Differences between black (people of African origin) and white (people of European origin) hypertensives are widely regarded as indisputable. Meta-analyses of findings from studies involving both American and non-American blacks and whites confirmed that blacks have a higher systolic and diastolic blood pressure (BP) than whites both at night and during the day.^1^ In the USA and South Africa, blacks had a higher prevalence of hypertension than whites in the same areas.^2^ The Centres for Disease Control recently published results from a study conducted from 1999 to 2002. The total prevalence of hypertension in the study group was found to be 28.6%. Of this percentage, 40.5% were blacks and 27.4% were whites.^3^ Blood pressure rises with age across all urban racial groups.^4^,^5^

Essential hypertension is a complex chronic disorder with a poorly understood pathogenesis. Renal sodium handling, ionic transport mechanisms, the renin-angiotensin-aldosterone system, vasoactive substances, the autonomic nervous system, diet, obesity, and environmental factors are all potentially implicated. This review will critically examine these factors to determine differences between black and white hypertensives.

## Renal sodium handling

In experimental models, kidney transplantation from a hypertensive to a normotensive rat causes hypertension in the recipient, and vice versa. This strongly suggests that hypertension may stem from the kidneys, since the previously normotensive rats became hypertensive. In humans undergoing renal transplantation there is an increased chance of developing hypertension if there is a history of hypertension in the donor’s family.^6^ Since the kidney is the main site for sodium handling,^7^ ethnic differences in sodium handling by the kidney may well be a causal factor of essential hypertension.

In response to high salt intake, a subgroup of individuals retains more sodium and undergoes a greater rise in blood pressure than others. This is termed salt sensitivity. For both normotensives and hypertensives, the blood pressure response of blacks to sodium loading is more salt sensitive,^8^-^10^ and there is a reduced ability to excrete a Na^+^ load, compared to whites.^11^ Brier and Luft^12^ suggest that sodium retention is perhaps an adaptive mechanism in people who originally came from a hot climate where salt was a scarce resource. As diets are now abundant in sodium, this mechanism would be maladaptive and would result in an increased extracellular fluid volume and hypertension, but this has proved difficult to demonstrate definitively.^13^,^14^

Several lines of evidence, however, support this hypothesis. It has long been recognised that there are differences in the renin-angiotensin-aldosterone system (RAAS) between blacks and whites. For the majority of normotensive and hypertensive South African blacks, plasma levels of renin and aldosterone are significantly lower than in whites.^15^,^16^ In the study by Rayner *et al.*,^16^ the lower plasma renin and aldosterone levels were not related to excess dietary intake of salt since there was no relationship between plasma renin activity (PRA) and sodium intake as assessed by spot urinary sodium/creatinine ratio. This suggests a dissociation between sodium intake and PRA,^16^ due to retention of sodium by the kidney and inhibition of the RAAS through negative feedback.

Additionally in black patients, plasma renin levels do not seem to increase in response to Na^+^ and volume depletion. 14,17 Sagnella also found that black hypertensives have a greater reduction in blood pressure in response to short-term Na^+^ restriction.^14^ These results suggest that the RAAS system in black hypertensives (and normotensives) is suppressed in response to sodium retention by the kidney. The most likely explanation for this is a complex interaction between genetic and environmental influences.

## Genetic determinants of salt sensitivity and hypertension in blacks

## Renal epithelial sodium channel (ENaC)

The renal epithelial sodium channel (ENaC) located within the collecting duct is one of the most important Na^+^ transport mechanisms in the overall control of Na+ balance, even though it only accounts for 5% of renal sodium absorption. It is responsible the final adjustment of sodium excretion, being the most distal site in the kidney for sodium reabsorption.

In rare instances, single polymorphic mutations have been found to cause hypertension and all of them are linked to increased sodium reabsorption by the kidney.^18^ In the rare autosomal-dominant disorder, Liddle’s syndrome, there is a molecular defect in the α- or β-subunits of the ENaC, which leads to sodium retention, hypokalaemia, hypertension, and suppression of both renin and aldosterone. This syndrome has similarities with hypertension in blacks, and mutations in the ENaC are strong candidate genes.^15^,^19^

Genetic variants of the ENaC subunits have been shown to be far more frequent in patients of African descent.^20^ The T594M mutation of the β-subunit of the ENaC, found by Baker *et al.*, is associated with low PRA and has been shown to be significantly more common in hypertensive than normotensive blacks.^21^ However, the prevalence of this polymorphism is very low, even in black subjects,^22^ and at least three studies have found no significant role for this variant in the development and severity of hypertension or the RAAS profiles of black patients.^22^,^23^

The G442V polymorphism is also frequently found in black patients and has been associated with decreased urinary aldosterone/K^+^ ratio – indicating increased ENaC activity. No association has been demonstrated with hypertension.^20^

Rayner *et al.*^24^ described the R563Q polymorphism of the β-subunit, which they found to be strongly associated with low-renin, low-aldosterone hypertension in South African black and mixed-ancestry patients. It is estimated that the prevalence of the R563Q mutation in the black population in the Western Cape is about 2% (pers commun – K Charlton). However it could not account for the low renin and aldosterone levels seen in normotensive blacks.^24^

On the other hand, Ambrosius and Pratt^25^ suggested that ENaC activity is lower in blacks due to Na^+^ retention occurring in the proximal tubule, caused by increased activity of the Na–K–2Cl co-transporter in the thick ascending limb of Henle or the thiazide-sensitive Na–Cl transporter in the distal convoluted tubule. This suppresses aldosterone secretion, which in turn suppresses ENaC activity. 25

## Renin-angiotensin-aldosterone system

The RAAS is a critically important endocrine system that regulates blood pressure and sodium balance. There are several candidate genes (ACE, angiotensinogen and aldosterone synthetase) in the RAAS that may potentially increase production of either angiotensin II or aldosterone, with suppression of renin, sodium retention and hypertension.

Tiago *et al.*^26^ found variants in the angiotensinogen gene (AGT) promoter region in a statistically significant number of South African black hypertensives with a body mass index (BMI) greater than 27 kg/m^2^, emphasising the importance of the environment on phenotypic expression. In addition, black hypertensives have an increased prevalence of aldosterone synthetase mutations compared to controls. Henderson *et al.*^27^ found data to suggest an association between the (-344)T allele of CYP11B2 (aldosterone synthetase) and an increased risk of hypertension in African−Americans. They found a similar association with the (-535)T allele of AGTR1 (angiotensin II type 1 receptor) gene. No association with ACE polymorphism and hypertension was found in the study population.^27^

## Ionic transport mechanisms

There are several lines of evidence to suggest that intra-cellular Na^+^ and Ca2^+^ concentrations are raised in both normotensive and hypertensive blacks compared to whites.^5^,^28^-^32^ However, these findings are not supported by all. Worthington *et al.*^5^ found that black hypertensives had normal levels of intracellular Ca2^+^. Blaustein proposed that the combination of raised intracellular Na^+^ and Ca2^+^ concentrations cause vasoconstriction of vascular smooth muscle, which raises peripheral vascular resistance and BP.^33^ Milne, on the other hand, hypothesised that the Na^+^ and Ca2^+^ overload would cause swelling of the vascular smooth muscle cells, reduction in luminal size and elevation of peripheral vascular resistance.^29^

The pathogenesis of the elevated intracellular Na^+^ and Ca2^+^ is probably related to a reduction in Na–K-ATPase activity in the cellular membrane. In several studies using erythrocytes and platelets from black and white normotensives and hypertensives, reduced activity was demonstrated in black hypertensives.^5^,^30^,^32^,^34^

There is some evidence that a digitalis-like factor may be responsible for reduced Na–K-ATPase activity in blacks, but its exact biological role is uncertain.^35^,^36^ Touyz *et al.*^30^ speculated that decreased intracellular Mg2^+^ may cause depression of Na–K-ATPase activity, since ATP requires Mg2^+^ in order to function. They also examined the diets of the urban blacks in their study and found them to be low in Ca2^+^ and Mg2^+^.^30^,^37^

Other perturbations in transport mechanisms have been observed in black hypertensives. Reduced activity of the Na–Li counter-transporter and increased Na–H counter-transport has been demonstrated.^32^,^38^,^39^ Increased Na–H counter-transport has been associated with a decreased expression of NHERF1 (an inhibitory protein of the Na–H counter-transporter).^40^

It is implied that these differences may account for the differences in intracellular ionic concentrations and thus for the differences in blood pressure between blacks and whites.^30^ However this has never been confirmed *in vivo* or *in vitro*, nor has the mechanism ever been sufficiently explained. Sagnella questioned the role of these pumps.^15^ Firstly, the Na–K-ATPase is also located in the basolateral membrane of the renal tubular epithelium, and is the driving force for Na^+^ reabsorbtion in the kidney. Reduced activity in the renal tubular epithelium is unlikely to cause increased Na^+^ reabsorption. Secondly, there is little known about the functional significance of the Na–Li counter-transporter and the effects of reduced activity.^15^

## Vasoactive substances

## Atrial natriuretic peptide

Atrial natriuretic peptide (ANP) is produced mainly in the cardiac atria in response to increased blood volume and atrial stretch.^41^ It results in increased urinary Na+ excretion. A reduction in ANP secretion or function could result in Na+ retention and salt-sensitive hypertension. In a study involving mice, researchers disrupted the proANP gene, with the result that the mice developed salt-sensitive hypertension.^42^

Human studies have shown that hypertensive blacks have low-to-normal plasma ANP levels.^43^-^45^ Kohno *et al*.^45^ found that Na^+^ loading increased plasma ANP more in salt-sensitive than salt-resistant patients. However, other studies have shown a lesser increase or decrease in plasma ANP levels in response to a high sodium diet in salt-sensitive compared to salt-resistant patients.^43^,^46^ In these patients, reduced atrial secretion of ANP during sodium loading could partly explain their reduced ability to excrete Na^+^ and the sodium-induced rise in blood pressure.^43^ In summary, these changes are interesting but do not establish causality with salt sensitivity or hypertension.

## Kallikrein

This enzyme is responsible for the synthesis of diuretic and renal vasodilating kinins^28^ and there is evidence suggesting that it may directly promote the conversion of inactive renin to active renin.^47^ Black normotensives and hypertensives have a lower urinary kallikrein than whites and this may be a possible mechanism for salt sensitivity.^48^,^49^

## Endothelin-1

Studies have shown that plasma concentrations of endothelin-1 (ET-1) are increased in hypertensive black patients.^50^ ET-1 is a vasoconstrictor, but also promotes Na^+^ retention^51^,^52^ and increases mitotic activity in vascular smooth muscle cells^53^ and cardiac myocytes.^54^ Campia *et al.*^55^ suggested that there is increased ET-dependent vasoconstriction in black hypertensives, compared to white hypertensives. They proposed this is due to an increased production or reduced clearance of ET-1 in blacks.

## Nitric oxide

Nitric oxide is an important regulator of vascular tone. Healthy black normotensives have reduced NO-mediated vasodilation, which could indicate impaired vascular smooth muscle relaxation, leading to increased vascular tone and possibly hypertension. 56,57

## Autonomic nervous system (ANS)

The ANS is a critical regulator of cardiac output, vascular tone and blood pressure. Little research has focused on the differences in the ANS between black and white hypertensives. No significant ethnic differences have been found in circulating catecholamines.^58^,^59^ However, it has been shown that blacks are more sensitive to the vasoconstrictor effects of noradrenaline and that this response is enhanced by a high-sodium diet.^60^ Studies have revealed lower levels of plasma dopamine β-hydroxylase in blacks, which may impair sodium excretion in the kidney.^61^,^62^

It has been demonstrated that blacks have a reduction in β_2_-receptor-mediated vasodilatation, which may lead to greater peripheral resistance.^63^,^64^ This may be related to ethnic differences in the occurrence of polymorphic variants in the β_2_-adrenoreceptor gene.^65^,^66^ However, it is uncertain how these variants are linked to the activity of the receptor.

## Dietary factors

The salt content in a westernised diet is several times more than the body’s physiological requirement. In the 1960s, Dahl measured the prevalence of hypertension among several population groups and found that blood pressure rises in direct proportion to salt consumption.^18^ However, several studies analysing dietary history and 24-hour salt excretion have failed to show a significant ethnic difference in Na+ intake between blacks and whites.^61^,^67^-^69^

An ethnic difference in potassium (K^+^) intake, however, has been suggested by several studies.^67^,^68^,^70^ Although urinary Na^+^ excretion was similar, both hypertensive and normotensive blacks excrete less K^+^ than whites. However, this disparity was not noted in all comparative studies.^61^,^71^ Recently, Charlton *et al.* studied dietary intakes of 325 black, white and coloured hypertensive and normotensive South African subjects.^72^ They found that white South Africans had a higher habitual intake of salt and calcium compared to their black and mixed-ancestry counterparts. All ethnic groups had excessive sodium intake, whereas potassium intakes in all groups were suboptimal. There were no dietary differences between hypertensives and normotensives.

The Dietary Approaches to Stop Hypertension (DASH) study^73^ revealed that a diet rich in potassium (fruits and vegetables), calcium (low-fat dairy products) and decreased total fat, together with sodium restriction significantly reduced BP in blacks. It is difficult to determine what part of the diet caused the decrease in BP. An increase in potassium may lower blood pressure^73^ but the mechanism is unclear. The results seem to reflect an interaction between the dietary cations, resulting in a decrease in BP. The DASH study therefore postulated that it is better to monitor salt intake together with levels of the other cations, than salt alone, in order to determine the exact effects on blood pressure.

As mentioned earlier, the diets of urban blacks were found to be low in Ca2^+^ and Mg2^+^. Alterations in Ca2^+^ uptake and metabolism have been implicated in the increased susceptibility to hypertension in blacks, and calcium supplementation has been known to cause a modest reduction in BP in some patients. Mg2^+^ depletion may result in reduced Mg-ATPase activity (see above).^28^

Recent analysis of the NHANES database supports the suggestion that inadequate calcium, potassium and magnesium intake was associated with hypertension.^74^

## Obesity

Obesity is a major risk factor for hypertension^75^ – people more than 20% over their ideal body weight have a two- to threefold higher risk of becoming hypertensive.^28^ Obesity is also an epidemic in the black community, especially among females^2^,^76^ and urban blacks.^2^ In a cross-sectional study by Sever *et al.*,^29^ BP correlated positively with BMI. Various mechanisms for the impact of obesity on BP have been suggested, among them, renal Na^+^ and water retention, increased activation of the sympathetic nervous system and of the renin-angiotensin system.^77^

There has been speculation that obese individuals have impaired response to and lower plasma levels of natriuretic peptide^77^ and there is limited evidence of plasma ANP levels failing to rise appropriately in obese patients after a saline load.^78^

The angiotensinogen gene is expressed in adipose tissue and variants of AGT could influence BP. Tiago *et al.*^26^ found variants in the AGT promoter region in a statistically significant number of South African black hypertensives with a BMI greater than 27 kg/m^2^. Up-regulation of this gene would accelerate angiotensin II (AII) and leptin production, stimulating the sympathetic nervous system (and thus vasoconstriction) and increasing plasma volume.^29^ However, an increase in AII is not necessarily reflected by its circulating plasma levels. The plasma level could be depressed as a result of the RAAS compensating due to increased sodium retention in black hypertensives.^29^

## The Barker hypothesis

The Barker hypothesis proposes that birth weight and adult BP are reciprocally regulated.^79^ The implication is that low-birth weight babies are more prone to hypertension later in life.^80^ The low birth weight is most likely caused by intra-uterine undernutrition. The proposed mechanisms for his hypothesis include reduced foetal kidney development, impaired endothelial development, increased sensitivity to glucocorticoids and a higher tendency to retain sodium due to reduced nephron number.^81^ There is evidence that in both the USA and South Africa, the prevalence of low birth weight in black babies is higher than in white babies.^82^,^83^

It seems probable that the Barker hypothesis could be a cause of hypertension in the developing world but it does not explain the discrepancy in prevalence of hypertension between rural and urban blacks.

## Urbanisation and stress

In South Africa, the average black person lives in worse socioeconomic circumstances than the average white. It has been found that the lower socio-economic group in South Africa has a higher prevalence of hypertension,^4^ suggesting a causal relationship. However, while the lower socio-economic group also had a lower level of education, it was found that for the same level of education, the prevalence of hypertension among blacks was still higher than among whites.^2^ This indicates that there are other contributing factors. The stress of residing in townships with poor living conditions, overcrowding and large families, together with a poor diet, alcoholism, poor access to healthcare and a decrease in physical activity could well contribute to hypertension. Chronic stress, brought on by unemployment and poverty is common in urban townships.

Stress is a known risk factor for hypertension and one would expect stress levels to be quite high in westernised populations, especially in urban blacks. Levy et al. found that blacks had increased sympathetic neuronal activity but insufficient to cause a sustained rise in BP,^84^ and similar circulating levels of catecholamines have been found in blacks and whites.^7^ This suggests that stress may not be an important factor in the differences between black and white hypertensives.

Cultural stress may play a role in the differing patterns of BP between races. Seedat uses the term ‘acculturation’ to describe the tendency for traditional black culture to be absorbed into western culture, and notes that the longer a particular culture has been ‘accultured’, the higher its prevalence of hypertension.^85^

It has been reported that the BP of an African black will rise within a few weeks of rural−urban migration^86^ and that the shift toward a western pattern of BP (ie, rising with increasing age) is associated with the embracing of a westernised diet and cultural stress.^4^ Inequalities in socio-economic status, lifestyle and access to healthcare in South Africa have contributed to the differences in complications of hypertension between blacks and whites.^87^

## Complications

Comparative studies have shown that black hypertensives have higher rates of malignant hypertension,^4^ and are more susceptible to hypertensive renal damage^85^ and renal failure^02^,^88^ than whites. Hypertensive blacks have a two-fold greater prevalence of left ventricular hypertrophy.^89^ This suggests that blacks have different adaptive cardiovascular changes in response to hypertension compared to whites. Congestive heart failure is more common among black hypertensives,^90^,^91^ which may be linked to the higher rates of left ventricular hypertrophy in blacks. It has also been demonstrated that coronary heart disease is less common in blacks of southern Africa than in whites.^90^,^91^

This may be due to the fact that blacks have lower fibrinogen, serum cholesterol and triglyceride levels and higher highdensity lipoprotein (HDL) levels than whites, which may be attributed to differing lifestyles and diet.^92^

Accessibility of healthcare, affordability and availability of drugs are limiting factors for black patients in South Africa. Many people living in urban informal settlements do not have ready access to screening for hypertension, and to antihypertensive treatment. Many people are ignorant of the dangers of untreated hypertension. These factors may partly explain some of the differences in complications between blacks and whites.

## Drug responses

The varying drug responses among hypertensive blacks and whites support the above-mentioned differences in hypertension between the two ethnic groups.

## Thiazide diuretics

Thiazide diuretics have been shown to cause a greater decrease in blood pressure in blacks than in whites.^93^,^94^ Since diuretics cause an increase in urinary sodium excretion and free water clearance,^95^ their effectiveness in blacks supports the claim that blacks have salt sensitivity.

## Calcium channel blockers

Studies have found that black hypertensives respond better to calcium channel blockers (CCBs) than whites.^96^,^97^ This may be related to differences in intracellular Ca2^+^ and Na^+^ concentrations and cellular calcium transport mechanisms, and the diuretic effect of calcium channel blockers.

## Beta-adrenoreceptor antagonists

Seedat found that black hypertensive patients have a poor response to β_1_-blockers.^98^ This may be due to the fact that a higher proportion of black hypertensives have low-renin hypertension, and an important mechanism of action of β_1_-blockers is inhibition of renin release.^95^

## Angiotensin converting enzyme (ACE) inhibitors

Black patients respond poorly to ACE inhibitors.^99^ However when ACE inhibitors are combined with a diuretic, blacks respond as well as whites.^98^ The poor response to monotherapy with ACE inhibitors is presumably due to suppression of the RAAS. Additionally, black hypertensives have a nearly threefold greater risk of angioedema compared to whites with the use of the ACE inhibitor enalapril.^100^

A recent authoritative meta-analysis by Brewster *et al.* supported these observations that blacks respond differently to antihypertensive medication.^101^

## Conclusion

Blood pressure is under the control of haemodynamic, cellular, genetic and hormonal factors and this review has proposed that a number of these factors could account for differences between black and whites.

Sodium appears to be the critical factor that signifies important differences between blacks and whites. In general, blacks are more salt sensitive than whites, and respond better to diuretics and CCBs than to ACE inhibitors or β-blockers as monotherapy. The mechanisms behind these differences are elusive, but are not due to dietary sodium intake. Although mutations in the ENaC, angiotensinogen gene and aldosterone synthetase could partly explain, it applies only to a small percentage of hypertensives. A far more common mutation would be required to account for the differences, but this has not as yet been found.

The review also suggests that environmental factors may be important in differences between blacks and whites. Tiago has recently elegantly demonstrated the interaction of obesity with an angiotensinogen mutation.

Differences in ionic transporters have been shown to be different between blacks and whites, but their significance is unknown. Causality with hypertension or salt sensitivity has not been established.

Currently, we believe it is reasonable to conclude that an acquired or inherited predisposition toward salt retention provides a basis for differences in blood pressure between blacks and whites. However, there are numerous potential confounding factors and while present research is continuing, antihypertensive treatment for patients needs to be carefully monitored, taking into account the individual’s lifestyle and ethnic group.
